# Teaching about the relativistic background of synchrotron radiation: some intriguing aspects with strong didactic implications

**DOI:** 10.1107/S160057752600603X

**Published:** 2026-07-15

**Authors:** G. Margaritondo

**Affiliations:** a Ecole Polytechnique Fédérale de Lausanne (EPFL), Switzerland; b Istituto Italiano di Tecnologia Morego Italy; Bhabha Atomic Research Centre, India

**Keywords:** synchrotron, Lorentz contraction, Doppler, speed of light, photon energy, relativity

## Abstract

Not fully grasping the concepts of relativity can lead to misunderstandings about the fundamental background of synchrotron emission. Here we deal with some intriguing cases, in which the relevant ‘speed of light’ is not the invariant *c* but the speed with respect to a moving object. This specifically affects two pillars of synchrotron radiation: the Lorentz length contraction and the Doppler shift. We propose a teaching strategy relying on new versions of simple ‘thought’ experiments. Besides putting synchrotron radiation on solid foundations, the approach amazingly leads to a unique link—described by Einstein as ‘remarkable’—between special relativity and quantum mechanics.

## Abstract 2

Explaining synchrotron radiation properties often requires using the speed of light with respect to a moving object like a detector, which may not be the invariant *c*. This surprising fact also leads to a rarely explored link between relativity and quantum mechanics.

## 1. Introduction

As is well known (Hwu & Margaritondo, 2021 ▸), the properties of synchrotron radiation are caused by special relativity, in particular by effects like length contraction, Doppler shift, Doppler beaming and time dilation. Therefore, teaching about them should be based on a correct use of relativity and its implications. However, subtle aspects are often missing in the students’ background, in particular of non-physicists.

We present here some intriguing examples directly relevant to synchrotron radiation. These are related to a simple question: what is the speed of light with respect to an object like a detector or a source, moving with constant speed *v* in the same direction? Many students would apply Einstein’s relativistic invariance (Einstein, 1905 ▸; Rafelski, 2017 ▸) and answer ‘‘*c*’’. This, however, is sometimes, perhaps surprisingly, incorrect.

To discover why, we must first deal with a semantic issue: what does ‘speed with respect to a moving object’ mean? There are two possible interpretations: first, the speed of light measured in an inertial reference frame in which the object is motionless. Second, the difference between the speed of light and that of the object, both measured in a reference frame in which the object moves with constant velocity.

In the first case, the answer ‘‘*c*’’ is correct due to the invariance principle. But this is not the quantity that one must use while deriving the properties of synchrotron radiation from phenomena like the Lorentz contraction and the Doppler shift. These require instead (Margaritondo, 1995 ▸) the second of the above interpretations—and the use of *c* ± *v* instead of *c* (the sign depends on the directions of the velocities).

## 2. Thought experiments

**Figure 1:**
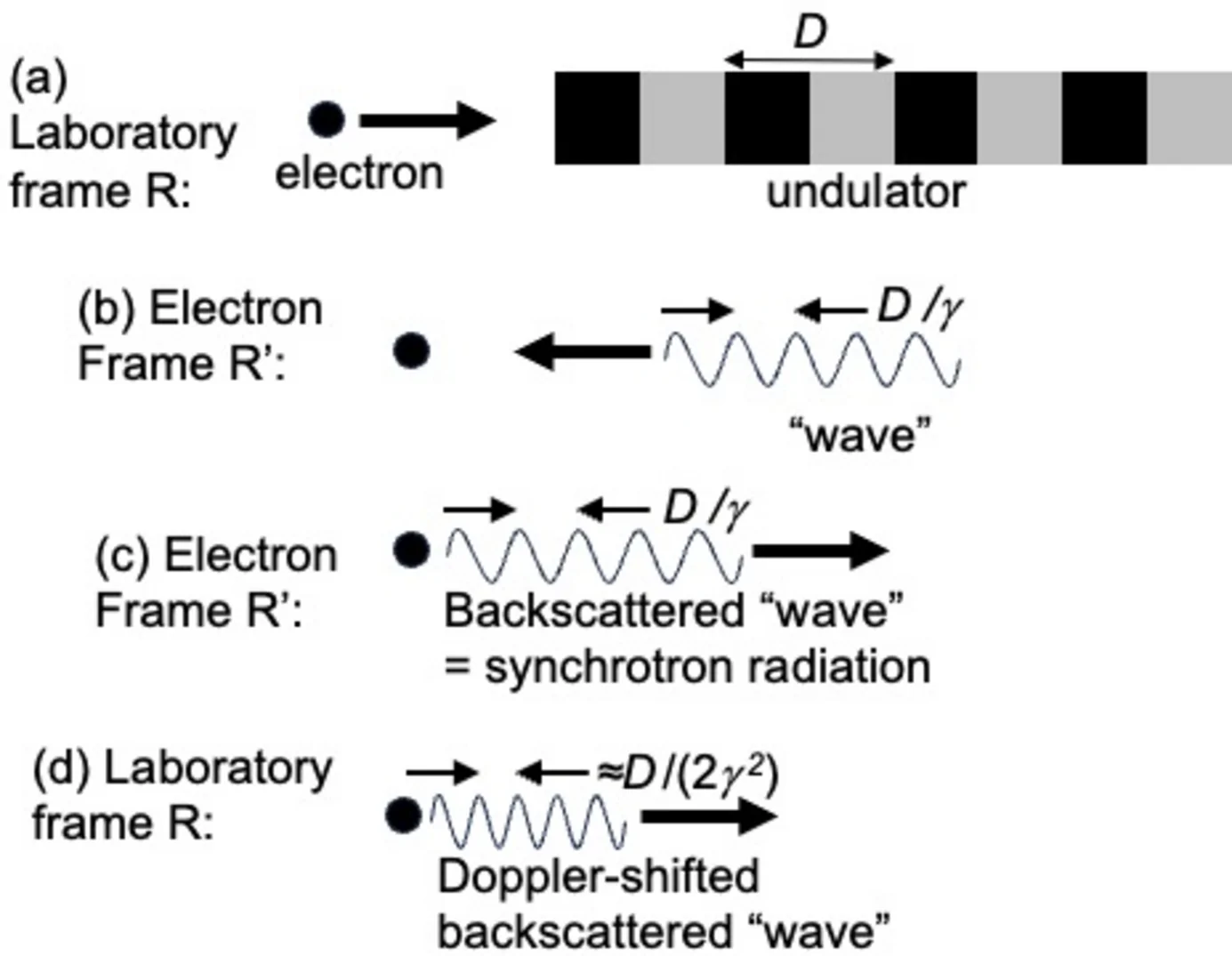
One of the simplest—yet conceptually correct—ways to explain synchrotron radiation properties, in this case the central emitted wavelength of an undulator of period *D*. (*a*) An electron approaches the undulator with relativistic speed. (*b*) ‘Seen’ by the electron, the undulator looks like a ‘wave’ with wavelength shrunk by the Lorentz contraction. (*c*) The ‘wave’ is backscattered by the electron: this is synchrotron radiation. (*d*) In the laboratory, the longitudinal (relativistic) Doppler shift further decreases by ∼1/(2γ) the wavelength, which becomes ∼*D*/(2γ^2^).

Let us, for example, analyze the central wavelengths emitted by an undulator of period *D*, as detected in the laboratory. Fig. 1 ▸ shows one of the simplest explanations. In the reference frame *R*′ [Fig. 1 ▸(*b*)] of an electron with a relativistic factor γ = 1/(1 − *v*^2^/*c*^2^)^1/2^, the periodic magnetic field of the undulator ‘looks’ similar to an electromagnetic wave of wavelength *D*′ = *D*/γ, the undulator period in its own (‘laboratory’) frame *R* shortened by the Lorentz contraction.

Backscattering this ‘wave’, the electron produces [Fig. 1 ▸(*c*)] synchrotron radiation, whose wavelength measured in the laboratory is decreased by the longitudinal Doppler shift [Fig. 1 ▸(*d*)], becoming ∼(*D*/γ)/(2γ) = *D*/(2γ^2^). This simple picture is validated by a full use of the Lorentz transformations, which shows, for example, that in the electron reference frame the magnetic field of the undulator becomes a combination of perpendicular and transverse electric and magnetic fields—indeed, like a propagating wave.

**Figure 2:**
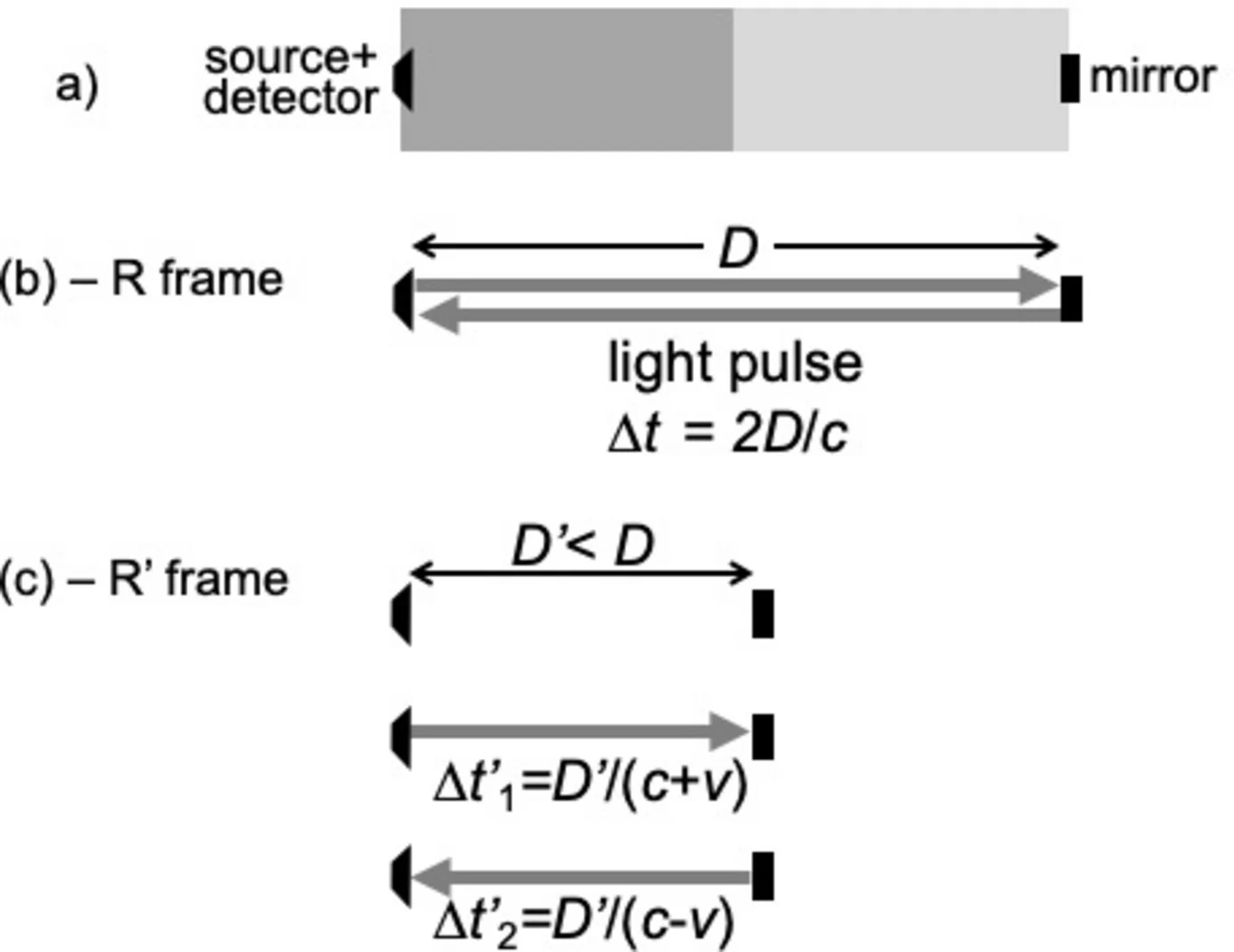
(*a*) A ‘thought’ experiment on light pulses to derive the Lorentz length contraction of the undulator period (Behroozi, 2014 ▸). (*b*) The experiment seen in the reference frame of the apparatus (and in particular of the undulator), *R*. (*c*) Measured in the frame *R*′ (the electron) which moves with speed *v* with respect to *R*, the distance *D*′ is Lorentz-contracted with respect to that measured in *R*, *D*. The figure illustrates the forward and backward motions of a light pulse in *R*′, from which the total time distance 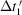 + 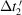 between ‘emission’ and ‘detection’ is derived.

The two relativistic ingredients of the above picture, Lorentz contraction and Doppler shift, can be derived with simple ‘thought’ experiments (Behroozi, 2014 ▸), such as that of Fig. 2 ▸ for the contraction, which uses a mirror plus a device that combines a source of light pulses and a detector, attached to the two ends of one undulator period. The experiment is observed in two inertial reference frames: *R* (undulator frame), where the apparatus is motionless, and *R*′ (electron frame), moving with respect to *R* with speed *v* in the same direction as the light pulses. Seen in *R*′, the experimental apparatus travels with speed −*v*.

In Figs. 2 ▸(*b*) and 2 ▸(*c*), *D* and *D*′ are the distances between the source–detector device and the mirror, measured in *R* and *R*′ and subject to the Lorentz contraction. In *R*, the time distance between emission and detection of a light pulse is
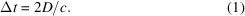
In *R*′, the corresponding time distance Δ*t*′ is given (Rafelski, 2017 ▸; Margaritondo & Rafelski, 2017 ▸) by the relativistic time transformation,
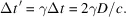
Δ*t*′ also equals the sum of 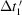 and 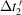, the times during which a light pulse travels from the source to the mirror and then from the mirror back to the detector,
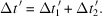
Assume now—erroneously—that the speeds to calculate 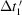 and 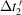 are both equal to the invariant *c*. We would get
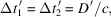

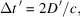

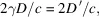
and
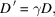
which is obviously wrong, corresponding in fact to an increase of *D*′ rather than to a contraction.

What was our mistake? We did not consider [Fig. 2 ▸(*c*)] the fact that the relevant speeds to calculate 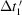 and 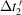 are *not* equal to *c* but to the differences between the speed of light and that of the source–mirror–detector apparatus, *v*,
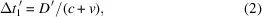

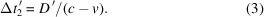
For example, during the time 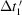 the distance over which the light pulse travels is shortened, because of the motion of the mirror, by 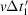, so that
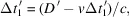
which gives equation (2). From equations (2) and (3) we get
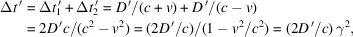

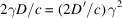
and
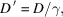
which is the correct Lorentz contraction.

**Figure 3:**
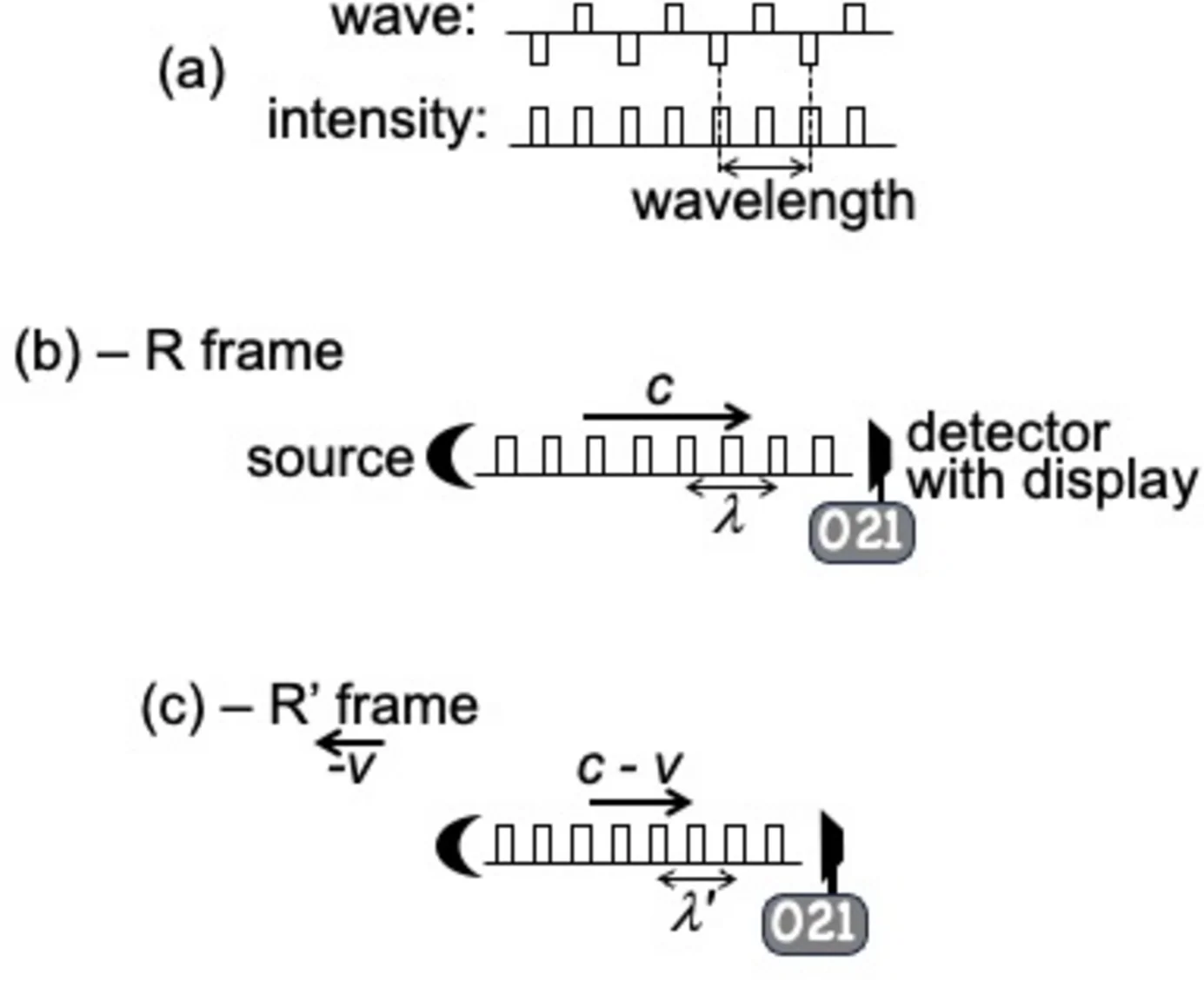
Analysis of the longitudinal Doppler shift with an experiment similar to Fig. 1 ▸ but with the source separated from the detector. The experiment detects a periodic series of light pulses. The detector is equipped with a numerical display showing the accumulated number of pulses. (*a*) A comparison of the wave with the corresponding intensity shows that each wavelength corresponds to two pulses. (*b*) In the frame *R*, source and detector do not move, whereas (*c*) in the frame *R*′, moving with speed −*v* with respect to *R*, source and detector travel with speed *v*. The accumulated number of ‘clicks’ shown by the display is the same in *R* and *R*′. This leads to the correct longitudinal Doppler shift formula, equation (6), only if one uses the speed difference *c* − *v* and not *c*.

A similar conclusion about relevant speeds is reached with the two ‘thought’ experiments that derive the longitudinal Doppler shift (Margaritondo & Rafelski, 2017 ▸) of Figs. 3 ▸ and 4 ▸. The first (Fig. 3 ▸) is a variant of the experiment of Fig. 2 ▸. However, source and detector are now two different devices and there is no mirror.

The emission consists of a periodic series of pulses traveling along the source–detector line. Note [Fig. 3 ▸(*a*)] that each wavelength corresponds to two intensity pulses. The detector is a pulse-counting device with a numerical display that shows the accumulated number of detected pulses.

Consider [Fig. 3 ▸(*b*)] the frame *R*, in which the source and the detector do not move. During a time interval Δ*t*, the detector ‘clicks’ *n* times, equal to twice the length *c*Δ*t* of the wave portion reaching the detector during Δ*t*, divided by the wavelength,
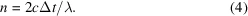
Adopt now [Fig. 3 ▸(*c*)] the reference frame *R*′ moving with speed −*v* with respect to *R*. In it, the source and the detector travel with speed *v*. The time interval Δ*t*′ in *R*′ that corresponds to Δ*t* is given, here again, by the relativistic transformation Δ*t*′ = γΔ*t*.

The motion of *R*′ causes the Lorentz contraction of the source–detector distance. Plus, it induces the longitudinal Doppler shift of the wavelength from λ to λ′, not explained by the Lorentz contraction alone. To correctly analyze λ′, we must evaluate the number *n*′ of detector clicks in *R*′ during Δ*t*′.

The length of the wave portion reaching the detector during Δ*t*′ is *V*′Δ*t*′, where *V*′ is the relevant velocity parameter in *R*′—which, as we shall see, is not *c*. Thus, the equivalent in *R*′ of equation (4) is
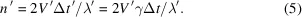
The number of clicks shown by the display must be, of course, the same for both reference frames: *n* = *n*′. So, equations (4) and (5) give
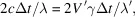

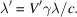
This result is equivalent to the formula for the Doppler shift in the longitudinal direction (Rafelski, 2017 ▸; Margaritondo & Rafelski, 2017 ▸),
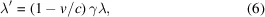
if
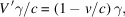

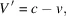
*i.e.* if we assume that the relevant velocity *V*′ is not *c* but the speed difference *c* − *v*, consistent with the conclusions derived above from Fig. 2 ▸.

**Figure 4:**
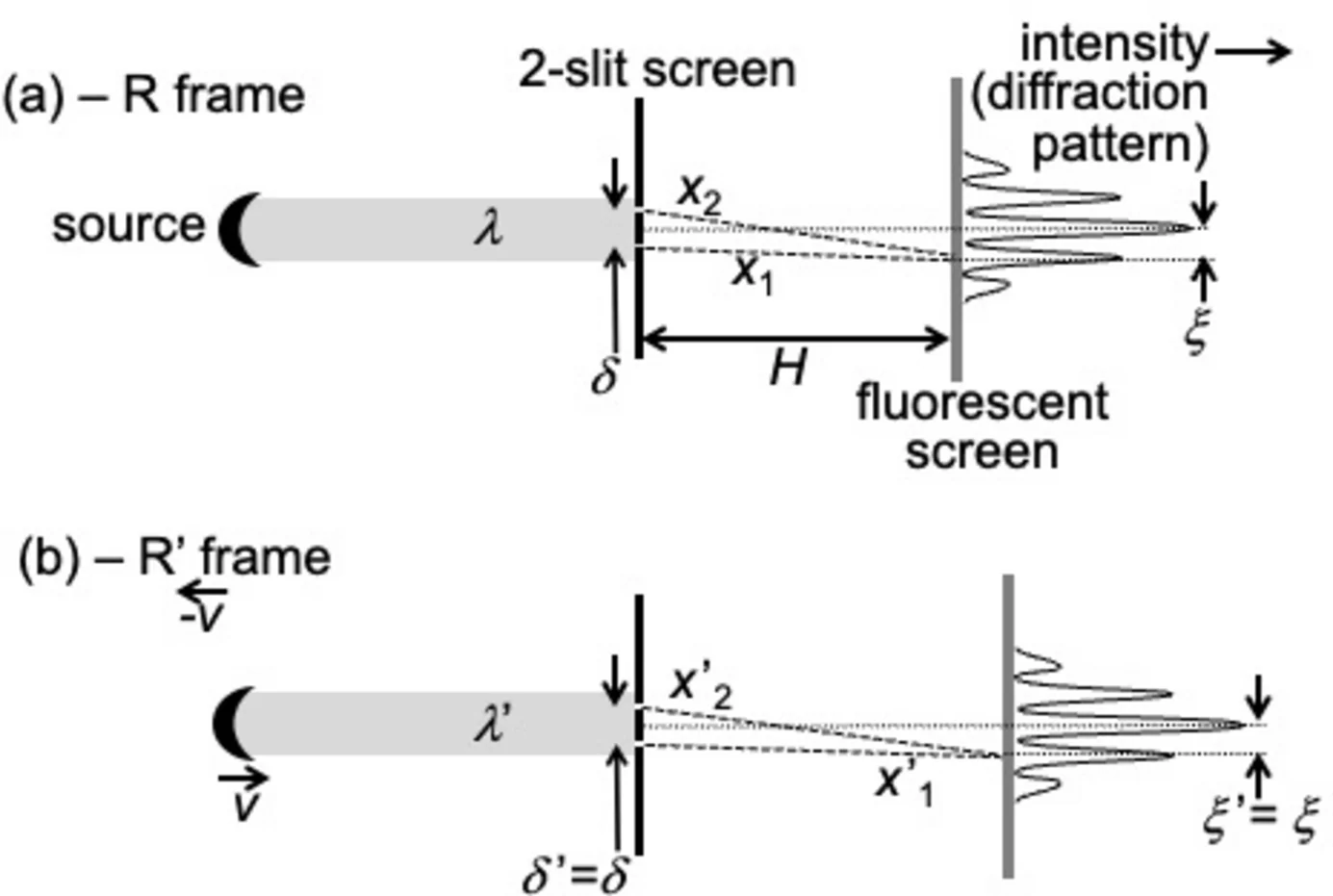
Another experiment on the Doppler shift, based on a specific phase-related phenomenon: two-slit diffraction. The results confirm again the conclusions about the relevant speed of light from the experiments of Figs. 2 ▸ and 3 ▸.

To further substantiate such conclusions, we can consider another way to derive the Doppler shift, Fig. 4 ▸. Its conceptual background is the relativistic principle that the relative motion of two inertial frames cannot be experimentally detected. In particular, it cannot be revealed by its consequences on wavefunctions (Margaritondo & Rafelski, 2017 ▸).

Thus, the phase of a wave must be the same in two inertial frames moving with respect to each other, otherwise phase-based effects like diffraction or interference could reveal their relative motion (note: the spatial and temporal phase gradients will nevertheless differ between the two frames). This fact can be directly used to derive the Doppler shift, as reported by Margaritondo & Rafelski (2017 ▸). However, the derivation can also be obtained by analyzing a specific phase-related effect, like the two-slit diffraction of Fig. 4 ▸.

Fig. 4 ▸(*a*) illustrates this phenomenon as seen in a reference frame *R* in which the entire apparatus is motionless, including the source. Let us call ξ the distance on the fluorescent screen between the first diffraction maxima and the central fringe. After passing through the slits, two waves arrive at one of these maxima with the paths *x*_1_ and *x*_2_. Constructive interference of simultaneously detected waves requires that
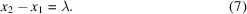
Calling δ the slit spacing, for a large slit–detector distance*H*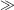 ξ,
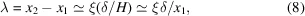

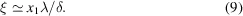
Consider now [Fig. 4 ▸(*b*)] a frame *R*′ moving with respect to *R* along the axis of the apparatus, with constant speed −*v*. The apparatus moves in *R*′ with speed *v*, and the source motion causes the Doppler shift from λ to λ′. The slit spacing δ is transversal and therefore not affected by the longitudinal motion of *R*′. But this motion does affect the paths 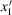 and 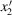, in two ways.

First, 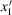 and 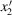 are Lorentz-contracted by a factor γ. Second, the two paths are extended by the motion of the fluorescent screen in *R*′. This motion takes place during a time interval ∼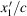 from the passage of the waves through the slits until their detection. Thus,
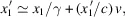

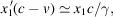

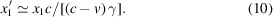
Therefore, the equivalent in *R*′ of equation (9) is
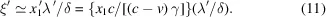
The relativistic principle of non-detectability of the relative motion of *R* and *R*′ requires the fringe positions on the fluorescent screen to be the same for both reference frames, ξ = ξ′. Therefore, equations (10) and (11) give
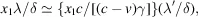

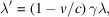
thus, we obtain once again the correct longitudinal Doppler shift formula of equation (6). The success of this derivation validates the adopted procedure, and in particular the use of equation (10), which is based on the difference (*c* − *v*).

Here again, whereas relativity requires the speed of light in a vacuum to be *c* in all inertial reference frames for plane waves, this does not apply to the differences of the speed of light with respect to those of moving objects like detectors. Note that this fact is consistent with the Lorentz velocity transformation between two inertial frames. Which changes *c* into *c*, whereas the speed differences are modified. For example, in the case of Fig. 3 ▸, the light–detector speed difference is *c* in *R* and it is transformed to *c* − *v* in *R*′, so it is *not invariant*.

## 3. A fundamental consequence

Is the use of speed differences rather than of *c* an important fact? Yes indeed: we have seen that it is essential if we want to correctly describe two fundamental relativistic phenomena—length contraction and the Doppler effect. But its impact extends beyond relativity, involving quantum physics and specifically the properties of photons.

Indeed, it can be used (Margaritondo, 1995 ▸) to derive the relation between photon energy and frequency. The approach is similar to that of Fig. 3 ▸, but the number of pulses is replaced by that of photons. Calling ρ the energy density of the light in *R*, Σ the active area of the detector and ε the photon energy, the number of photons counted during Δ*t* is ρΣ*c*Δ*t*/ε.

Changing the reference frame to *R*′, *c* is replaced (Margaritondo, 1995 ▸) by *c* − *v*, Σ is invariant, Δ*t* changes to Δ*t*′ = γΔ*t*, ε becomes ε′, and ρ is transformed into ρ′ = γ^2^(1 + *v*/*c*)^2^ρ. Thus, the fact that the number of clicks is the same in *R* and in *R*′ requires that
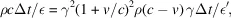

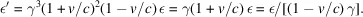
This formula is the reciprocal of equation (6), implying that the photon energy transforms as 1/λ, *i.e.* as the frequency ν =*c*/λ. And therefore it is proportional to ν: ε = *hν*.

Einstein presented (Einstein, 1905 ▸) an intriguing comment about this fact: ‘*It is remarkable that the energy and the frequency of a light complex vary with the state of motion of the observer in accordance with the same law*’. Why remarkable?

To answer, one should note that shortly before the article on relativity Einstein had published the photon hypothesis (Einstein, 1905 ▸). This was *not* based on the photoelectric effect (as many erroneously believe) but on elegant thermodynamics arguments—and was astonishingly revolutionary.

This publication was extremely risky for a recently graduated PhD with no academic job. How could he find the necessary courage? Most likely, the ‘remarkable’ proportionality of photon energy and frequency—that he had independently discovered—inspired him. A proportionality that is related, as we have seen here, to the fact—perhaps surprising—that the ‘speed of light with respect to a moving object’ is not necessarily *c*.

## 4. Didactic use and messages

The teachers should of course feel free to use the above material according to their own plans, but we would like to propose some suggestions. The arguments about the non-invariance of light speed differences can be presented stressing the risks of using relativistic notions without fully digesting them. And stimulating the students to re-visit their relativity courses.

In addition, the different ‘thought’ experiments can be used to understand the corresponding relativistic effects used to treat synchrotron radiation rather than just taking formulas.

Finally, the link between relativity and quantum physics can be used to put in the correct historical light the courage of young Einstein in proposing the photon, a fact that should inspire students not much younger than he was.

## References

[bb1] Behroozi, F. (2014). *Phys. Teach.* **52**, 410–412.

[bb2] Einstein, A. (1905). *Annal. Phys.* **322**, 891–921; *Ann. Phys.* **17**, 132–147.

[bb3] Hwu, Y. & Margaritondo, G. (2021). *J. Synchrotron Rad.* **28**, 1014–1029. 10.1107/S1600577521003325 PMC8127362 33950010

[bb4] Margaritondo, G. (1995). *Eur. J. Phys.* **16**, 169–171.

[bb5] Margaritondo, G. & Rafelski, J. (2017). *J. Synchrotron Rad.* **24**, 898–901. 10.1107/S160057751700769X PMC5493028 28664897

[bb6] Rafelski, J. (2017). *Relativity Matters: From Einstein’s E = mc^2^ to Laser Particle Acceleration and Quark-Gluon Plasma.* Springer.

